# Correction of Gradient Nonlinearity Bias in Apparent Diffusion Coefficient Measurement for Head and Neck Cancers Using Single- and Multi-Shot Echo Planar Diffusion Imaging

**DOI:** 10.3390/cancers17111796

**Published:** 2025-05-28

**Authors:** Ramesh Paudyal, Alfonso Lema-Dopico, Akash Deelip Shah, Vaios Hatzoglou, Muhammad Awais, Eric Aliotta, Victoria Yu, Thomas L. Chenevert, Dariya I. Malyarenko, Lawrence H. Schwartz, Nancy Lee, Amita Shukla-Dave

**Affiliations:** 1 Department of Medical Physics, Memorial Sloan Kettering Cancer Center, New York City, NY 10065, USA; 2 Department of Radiology, Memorial Sloan Kettering Cancer Center, New York City, NY 10065, USA; 3 Department of Radiology, University of Michigan, Ann Arbor, MI 48109, USA; 4 Department of Radiation Oncology, Memorial Sloan Kettering Cancer Center, New York City, NY 10065, USA

**Keywords:** diffusion-weighted magnetic resonance imaging, gradient nonlinearity correction, multiple b-value, apparent diffusion coefficients

## Abstract

**Background/Objectives:** This work prospectively evaluates the vendor-provided Low Variance (LOVA) apparent diffusion coefficient (ADC) gradient nonlinearity correction (GNC) technique for primary tumors, neck nodal metastases, and normal masseter muscles in patients with head and neck cancers (HNCs). **Methods**: Multiple b-value diffusion-weighted (DW)-MR images were acquired on a 3.0 T scanner using a single-shot echo planar imaging (SS-EPI) and multi-shot (MS)-EPI for diffusion phantom materials (20% and 40% polyvinylpyrrolidone (PVP) in water). Pretreatment DW-MRI acquisitions were performed for sixty HNC patients (n = 60) who underwent chemoradiation therapy. ADC values with and without GNC were calculated offline using a monoexponential diffusion model over all b-values, relative percentage (r%) changes (Δ) in ADC values with and without GNC were calculated, and the ADC histograms were analyzed. **Results**: Mean ADC values calculated using SS-EPI DW data with and without GNC differed by ≤1% for both PVP20% and PVP40% at the isocenter, whereas off-center differences were ≤19.6% for both concentrations. A similar trend was observed for these materials with MS-EPI. In patients, the mean rΔADC (%) values measured with SS-EPI differed by 4.77%, 3.98%, and 5.68% for primary tumors, metastatic nodes, and masseter muscle. MS-EPI exhibited a similar result with 5.56%, 3.95%, and 4.85%, respectively. **Conclusions**: This study showed that the GNC method improves the robustness of the ADC measurement, enhancing its value as a quantitative imaging biomarker used in HNC clinical trials.

## Simple Summary

Diffusion-weighted magnetic resonance imaging (DW-MRI) is a widely used noninvasive technique for the characterization of tumor cellularity in head and neck cancers (HNC). The new vendor-provided Low Variance (LOVA) apparent diffusion coefficient (ADC) gradient nonlinearity correction (GNC) technique improves the accuracy of the ADC values. This GNC method was initially tested for breast cancer patients. The present study aimed to investigate the performance of the LOVA GNC method for HNC patient ADC. The GNC-corrected mean ADC values of primary tumors and metastatic neck nodes were lower than the uncorrected ADC values. The shift in ADC histograms for primary tumor and metastatic nodes was observed with and without the application of GNC. The results showed that implementing GNC improves the ADC measurements.

## 1. Introduction

Diffusion-weighted (DW)-MRI-derived apparent diffusion coefficient (ADC) measurement has shown promise in head and neck cancer (HNC) [1,2,3,4,5]. Since the freedom of motion of water molecules is hindered by interactions with other molecules and cellular barriers, water molecule diffusion abnormalities can reflect changes in tissue organization at the cellular level [6,7,8,9]. These tissue diffusion properties can be quantified with the monoexponential modeling of the DW-MRI signal decay as a function of diffusion sensitivity (b-value). Microstructural changes affect the (hindered) motion of water molecules and consequently alter the water ADC [10,11,12,13]. ADC, a surrogate of tumor cellularity, allows the evaluation of treatment response in HNC [14,15,16,17,18,19]. Vandecaveye et al. found that the ADC changes (ΔADC) between pretreatment and after the chemoradiation therapy (CRT) of lesions with later tumor recurrence were significantly lower than lesions with complete remission for both primary lesions (*p* < 0.0001) and adenopathies (*p* = 0.003) [16]. Paudyal et al.’s HNC study results exhibited an increasing trend in ADC at each intra-treatment week when compared with pretreatment in the complete response group (*p* < 0.003) who received CRT [20]. For quantitative ADC application, the measured changes need to be analyzed with respect to confidence intervals determined by precision and bias [21,22,23].

Previous studies have demonstrated that the ADC measurement can be biased by spatially-dependent b-value due to gradient nonlinearity (GNL) [24,25,26,27], particularly for anatomy offset from the MRI scanner’s isocenter. The scientific yield of imaging trials has been diminished by the confounding presence of significant platform-dependent heterogeneity and systematic spatial bias in DW-MRI [28,29]. This instrumental bias is patient-independent and predictable through gradient system parameters but is distinct from geometric distortions routinely corrected by MRI vendors. Because this bias is dependent on system gradient design, it increases technical variability across scanners [27,30]. A method has been developed and implemented across three dominant MRI vendors to eliminate platform-dependent GNL bias in ADC for clinical DW-MRI applications [31]. This GNL correction (GNC) is tailored to DW-MRI acquisition based on three orthogonal gradient directions and has allowed improvements in both absolute accuracy and multiplatform ADC reproducibility [31].

The feasibility and effectiveness of a retrospective GNC implementation in the clinical setting were tested with quantitative quality control phantom and in vivo for subjects from the ACRIN 6698 breast cancer therapy response trial scanned on different MRI systems using single-shot (SS) echo planar imaging (EPI) DW-MRI [32]. The GNC ADC correction was applied to trace DW-MRI using system-specific gradient-channel fields derived from vendor-provided spherical harmonic tables. Across studied trial subjects, GNC improved the accuracy of ADC histogram metrics. Recently, the GNC method has been implemented on clinical scanners via an academic-industry partnership with the three predominant MRI vendors. The ongoing GNC integration with advanced DW-MRI acquisition methods, including multi-shot (MS)-EPI, that are increasingly implemented on clinical scanners requires validation [33,34,35,36]. Previous studies have emphasized that implementing GNC is crucial for improving the accuracy of ADC, ensuring consistent ADC values across different imaging sessions and scanners. This is particularly important in longitudinal studies, where consistent and precise measurements are essential for evaluating treatment responses over time. In the present study, we aimed to prospectively evaluate the vendor-provided Low Variance (LOVA) ADC GNC technique and integrate it with the clinical study workflow to improve the accuracy of the ADC values measured for primary tumors, neck nodal metastases, and normal masseter muscles in patients with HNC.

## 2. Materials and Methods

### 2.1. ADC Phantom Data Acquisition and Analysis

GNC was initially tested for the monoexponential diffusion phantom materials polyvinylpyrrolidone (PVP) in water within the reference phantom with calibrated ADC values [37]. The 20% and 40% PVP material vials (20 mL scintillation glass) were placed in a 1 L jar filled with water (in a stack with a 2.0 cm offset from the center) [37]. The phantom jar included an alcohol thermometer to record the temperature (20 ± 0.2 °C). The scans were performed at ambient temperature on a 3.0 T MRI (Elition, Philips Healthcare, Best, The Netherlands) using a 16-channel head coil with a phantom jar placed at a magnet isocenter and off-center (12 cm). DW-MRI images of the phantom were acquired using single-shot echo planar imaging (SS-EPI) and multi-shot (MS) (2-shot)-EPI sequences with nine b-values (i.e., b = 0, 100, 200, 500, 800, 1000, 1500, 2000, and 2500 s/mm^2^) and the following parameters: repetition time (TR) = 5000 ms, echo time (TE) = minimum (61 ms), number of averages (NA) = 1, SENSE = 2, acquisition matrix = 128 × 128, field of view (FOV) = 230 mm^2^, number of slices (NS) = 20, slice thickness = 4 mm, with diffusion encoding in 3 orthogonal directions. The total acquisition time for the multiple b-value DW-MRI data acquisition was ~3–4 min.

For real-time processing, the LOVA ADC GNC technique was applied during acquisition. This study generated two DW-MRI datasets with and without GNC options. On both ADC maps, a 200 mm^2^ region of interest was placed on PVP20% and PVP40% phantom vials using ITK-SNAP. These two PVP reference solutions represent ADC in human tissue ranges and are also available in the Diffusion “NIST/QIBA” Phantom, with ADC values reported in previous studies [37].

### 2.2. HNC Patient

The institutional review board approved this prospective study, which was compliant with the Health Insurance Portability and Accountability Act. We obtained written informed consent from all 60 eligible HNC patients (Table 1) who underwent pretreatment MRI scans between December 2021 and March 2024 and were treated with definitive CRT.

### 2.3. HNC DW-MRI Data Acquisition

MRI protocol consisted of clinical standard-of-care (SOC) T1/T2 weighted (w) imaging followed by a multi-b-value DW-MRI on a 3.0 T scanner (Elition, Philips Healthcare, Best, The Netherlands) using a neurovascular phased-array coil. The multiple b-value DW images were acquired using SS-EPI and MS-EPI sequences. SS-EPI was acquired for all 60 patients and MS-EPI for a subset of 29 HNCs. The data acquisition parameters were as follows: field of view (FOV) = 20–24 cm, matrix = 128 × 128, slices = 10–12, slice thickness = 5 mm, number of excitation (NEX) = 2, and 10 b-values = 0, 20, 50, 80, 200, 300, 500, 800, 1500, and 2000 s/mm^2^. For SS-EPI, TR/TE = 4000/80 (minimum) ms, and, for MS-EPI, TR/TE = 3000/66 (minimum) ms; other parameters were the same as mentioned above.

### 2.4. HNC Regions of Interest Contouring and Data Analysis

A neuro-radiologist delineated regions of interest (ROI) for SS-EPI and MS-EPI acquisitions on primary tumors (n = 38 and 19), neck nodal metastases (n = 55 and 28), and the normal masseter muscle (n = 44 and 24) on the DW image (b = 0 s/mm^2^) using ITK-SNAP. The primary tumors and neck nodal metastases were evaluated prospectively on T2w images and the highest b = 2000 s/mm^2^ before contouring on DW images. All DW-MRI data analysis was performed using in-house software MRI-QAMPER v3 (Quantitative Analysis Multi-Parametric Evaluation Routines) written in MATLAB v2023 (MathWorks, Natick, MA, USA) [38]. ADC values were calculated, and ADC maps were generated using a monoexponential model [39], including all b-values, as follows:

(1) $$S_{b}=S_{0}e^{-b\times ADC}$$

where S_b_ and S_0_ are the signal intensities with and without diffusion weighting, and b is the diffusion-weighting factor (s/mm^2^). Both with and without the GNC DW-MRI-signal intensities data were used for calculating ADC values.

The relative percentage (r (%)) change (Δ) in mean ADC values with and without GNC was calculated as follows:

rΔADC (%) = (ADC − ADC_GNC_)/ADC_GNC_ × 100 (2)

where ADC_GNC_ and ADC represent the ADC values measured with and without GNC, respectively.

ADC histogram analysis was conducted using the ADC maps generated from the ROI defined for the primary tumor, neck nodal metastases, and masseter muscle.

### 2.5. Statistical Analysis

ADC mean, standard deviation (SD), skewness, and kurtosis values with and without the LOVA ADC GNC were reported and compared using a Wilcoxon matched-pairs signed-rank (WSR) test for the SS-EPI and MS-EPI techniques. Bland Altman analysis assessed the agreement between ADC measurements with and without GNC for both SS-EPI and MS-EPI. A *p*-value < 0.05 was considered statistically significant.

## 3. Results

### 3.1. Phantom

ADC values for reference vials PVP20% and PVP40% measured with SS-EPI and MS-EPI are given in Table 2. Mean ADC values calculated using SS-EPI data with and without GNC differed by ≤1% for PVP20% and PVP40% at isocenter, whereas off-center differences were ≤18.8% for both concentrations. A similar trend of ≤1% and ≤19.6% was observed for PVP20% and PVP40% with MS-EPI, respectively.

### 3.2. Patient

The ADC histograms in Figure 1 and Figure 2, acquired using SS-EPI and MS-EPI, illustrate the effect of LOVA ADC GNC on the ADC measurements for ROI, i.e., primary tumors and neck nodal metastases from two representative patients with HNC. All voxels within the tumors were used for the histogram plots. In Figure 1, narrower ADC histograms were observed for both the primary tumor and node after GNC, while, without GNC, there was a significant broadening of the respective histograms using SS-EPI.

In Figure 2, the ADC histogram exhibits comparable width and minimal shift before and after GNC for both the primary tumor and metastatic node using MS-EPI. In addition, MS-EPI (Figure 2) exhibited enhanced spatial resolution and image quality compared to SS-EPI (Figure 1), particularly in regions with complex tissue structures in the head and neck.

A comparison of representative ADC histograms from ADC maps of the masseter muscle with and without GNC for MS-EPI is shown in Figure 3 from the same patient in Figure 2. All voxels within the ROI in the representative slice were used for the histogram plots. ADC histograms exhibited similar widths and minimal shift before and after GNC. A similar trend was observed for the masseter muscle with SS-EPI.

Table 3 summarizes the ADC mean (Figure 4), SD, skewness, and kurtosis values measured in HNC patients at pretreatment for primary tumors, nodes, and masseter muscle using SS-EPI and MS-EPI. For SS-EPI, mean ADC values with and without GNC exhibited significant differences for three ROI, i.e., primary tumors, metastatic nodes, and masseter muscle (*p* < 0.05). Mean ADC values obtained with MS-EPI data acquisition exhibited a similar trend for these three ROI (*p* < 0.05). The mean rΔADC (%) values measured with SS-EPI differed by 4.77%, 3.98%, and 5.68% for primary tumors, metastatic nodes, and masseter muscle. MS-EPI exhibited a similar trend of 5.56%, 3.95%, and 4.85%, respectively. The ADC kurtosis and skewness depict the changes in the peakedness and the tails of the ADC histograms. For example, the mean ADC skewness measured using SS-EPI changes by 13.3%, 7.1%, and 11.1% for the primary tumors, metastatic nodes, and masseter muscle, respectively, and a similar trend was observed for MS-EPI. 

Figure 5 and Figure 6 show the Bland–Altman plots and the mean percent difference (bias) of ADC measurements for primary tumors, metastatic nodes, and masseter muscle, which were 3.4%, 3.2%, and 5.8% with SS-EPI. Meanwhile, for MS-EPI, biases were 5.4%, 3.9%, and 5.0%, respectively (Table 4).

## 4. Discussion

DW-MRI is primarily used for lesion detection and characterization in HNC [4,40]. The ADC is a quantitative imaging biomarker used for CRT response assessment in HNC, reflecting changes in the tumor cellularity [1,5,41,42]. Utilizing DW-MRI as a diagnostic tool in clinical trials and response monitoring without correcting the spatial variation of b-value introduces confounding bias in ADC measures for large anatomical variations across patients and during longitudinal MRI exams [28,29]. Thus, it is necessary to implement and optimize DW-MRI sequence parameters as well as new practical correction methods of DW bias, such as GNC across the multisite and MR scanners for reliable and reproducible ADC measurement [32,43]. The phantom results in this study indicated that the ADC values with both SS-EPI and MS-EPI data acquisition techniques were similar; however, the phantom’s location caused spatial variation in b-values, affecting ADC calculations. In the representative figures, the HNC patient ADC histograms were narrower after GNC with SS-EPI, while the histograms showed comparable width and minimal shift with and without GNC for MS-EPI. The mean ADC values for MS-EPI were slightly higher than the SS-EPI, with comparable bias for the primary tumor and the metastatic lymph nodes. In contrast, mean ADC values derived using both techniques were similar for the masseter muscle with and without GNC was seen in the representative figure. These results demonstrate that incorporating GNC-corrected ADC values into clinical practice can enhance diagnostic accuracy and provide robust quantitative metric values for the evaluation of treatment response, specifically reducing variability and improving the comparability of results across the different MRI systems.

In the present study, the mean ADC values, derived from DW-MRI data acquired using SS-EPI, were 0.75, 0.87, and 1.15 for primary tumors, metastatic nodes, and masseter muscle without GNC, which were consistent with our previous results for HNC [33]. SS-EPI readouts are prone to substantial image distortions near magnetic field inhomogeneities. Advancements have been made in DW-MRI acquisition, including head and neck and many other organs [44,45,46,47,48,49], for example, the multiplexed sensitivity-encoding (MUSE) method, which uses the conventional sensitivity encoding (SENSE) technique [50,51] to measure the motion-induced phase variations among multiple shots and then performs joint unaliasing from all the shots. Konar et al. reported ADC values of 1.620 × 10^−3^ (mm^2^/s) and 1.584 × 10^−3^ (mm^2^/s) for the masseter muscle using the SS-EPI and MUSE-EPI for a paired b-value of [0, 1000] s/mm^2^ derived from DW-MRI data acquired on a 3.0 T General Electric Health Care MRI scanner [33]. The present study was performed on a Philips Healthcare 3.0 T scanner, and the ADC values for masseter muscle were 1.15 ± 0.41 × 10^−3^ (mm^2^/s) and 1.09 ± 0.27 × 10^−3^ (mm^2^/s) for SS-EPI and MS-EPI without GNC, respectively, acquired using 10 b-values. The lower ADC values could be due to the noise floor bias. Recently, Aliotta et al. showed that MS-EPI acquisition exhibited improved geometric distortion and that the MS-EPI sequence available on a Philips 3.0 T scanner offers an appealing middle-ground with improved geometric fidelity but superior efficiency and in vivo ADC quantification [52]. Reduced distortion with MS readout breaks up acquisitions into multiple, shorter blocks that are individually less susceptible to distortions or blurring [36,52,53]. Similarly, our study showed higher SNR for MS-EPI compared to SS-EPI for HNC.

Substantial ADC bias for HNC location offset from the magnet isocenter is also observed and is primarily attributed to spatially nonuniform diffusion weighting (b-value), owing to system-dependent gradient nonlinearity [28,29]. Malyarenko et al. exhibited the feasibility of centralized retrospective ADC correction for DW-MRI acquired as part of a multiplatform breast cancer imaging trial [32]. The notable GNC impact on tumor ADC histogram percentiles promises improvement in accuracy and reproducibility for diagnostic and prognostic thresholds sought for quantitative breast cancer treatment response assessment [32]. Our study in HNC shows similar results for SS-EPI: the ADC histograms with the LOVA ADC GNC method were narrower for both primary tumors and metastatic nodes.

Our study has a few limitations. To reduce discomfort and scan time for clinical HNC patients, the MS-EPI was performed on a subset of this population. This analysis focused on 3.0 T imaging performed on a Philips scanner. The tradeoffs of distortion and SNR are different for lower field strengths such as 1.5 T. While 3.0 T MRI provides higher resolution and better SNR, it is more susceptible to artifacts, especially in areas with high magnetic susceptibility like the neck, due to complex structures and interfaces such as bone, skull base, and fatty tissue [54,55,56]. Lastly, the performance of the GNC method was not compared between multiple MRI vendors and different field strength scanners, as this was beyond the scope of this study.

## 5. Conclusions

This present study showed promising results that implementing the GNC method improves the robustness of the ADC measurement, thereby enhancing its value as a quantitative imaging biomarker used in HNC clinical trials.

## Figures and Tables

**Figure 1:**
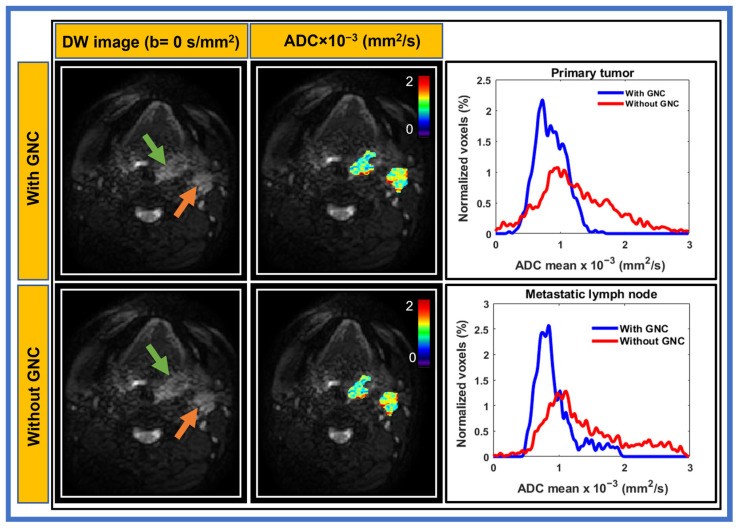
Examples of diffusion-weighted images (b = 0 s/mm^2^) acquired using single-shot echo planar imaging and apparent diffusion coefficient (ADC) maps of primary tumor (green arrow), and metastatic lymph node (orange arrow) with and without gradient nonlinearity correction (GNC) from a representative head and neck cancer patient (53-year-old male). The ADC maps exhibiting lower ADC values after GNC both in primary tumor and lymph node and the histograms were narrower for both.

**Figure 2:**
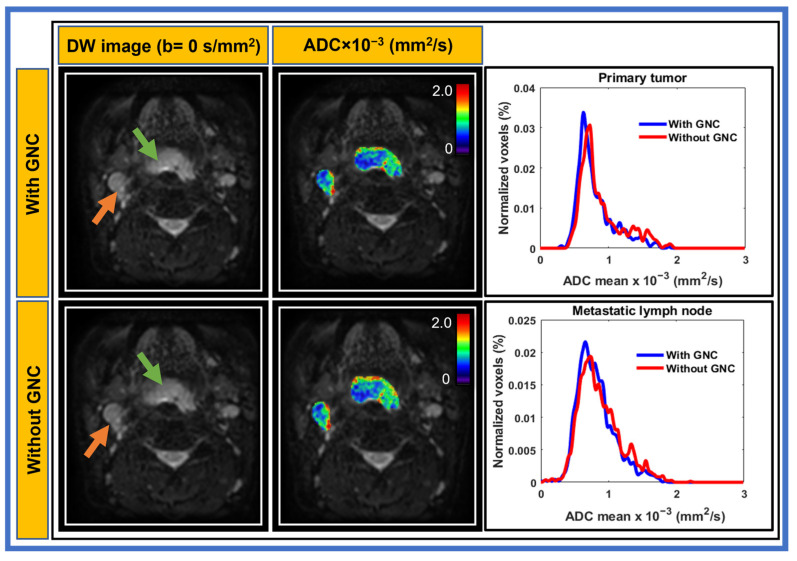
Example of diffusion-weighted- images (b = 0 s/mm^2^) acquired using multi-shot echo planar imaging and apparent diffusion coefficient (ADC) maps of primary tumor (green arrow), and metastatic lymph node (orange arrow) with and without gradient nonlinearity correction (GNC) from a representative head and neck cancer patient (55-year-old male). The ADC histograms were of similar width and with minimal shift for both.

**Figure 3:**
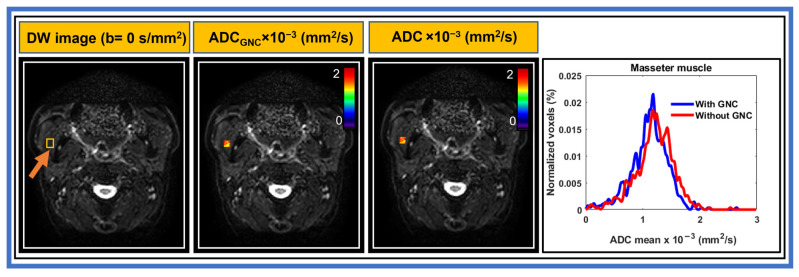
Example of diffusion-weighted MR images (b = 0 s/mm^2^) acquired using multi-shot echo planar imaging (EPI) and apparent diffusion coefficient (ADC) maps of masseter muscle (rectangle in a yellow color) with and without gradient nonlinearity correction (GNC) from a representative head and neck cancer patient (55-year-old male). The ADC maps exhibiting slightly lower ADC values after GNC. The histograms were of similar width and with minimal shift.

**Figure 4:**
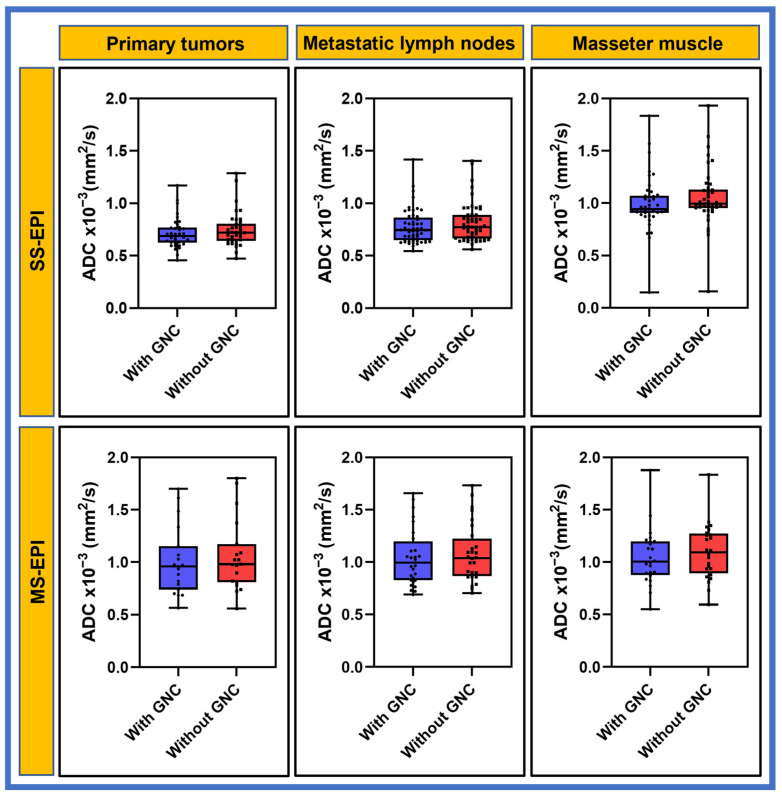
Box plots comparing the mean apparent diffusion coefficient (ADC) values with and without gradient nonlinearity correction (GNC) for primary tumors, metastatic nodes, and masseter muscle obtained from the single-shot (SS) echo planar imaging (EPI) and multi-shot (MS) EPI. The horizontal line in box represents the median. Significant differences were found between with and without GNC for three regions of interest. ADC values with and without from both SS-EPI and MS-EPI techniques were significantly different (*p* < 0.05).

**Figure 5:**
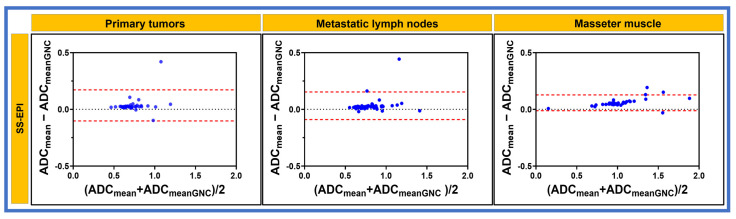
Bland–Altman plot exhibiting agreement between the measurements of mean apparent diffusion coefficient (ADC × 10^−3^ mm^2^/s) values obtained with and without gradient nonlinearity correction (GNC) from single-shot echo planar imaging (EPI) for primary tumors, metastatic lymph node, and masseter muscle. The dash lines (red) are the 95% limits of agreement.

**Figure 6:**
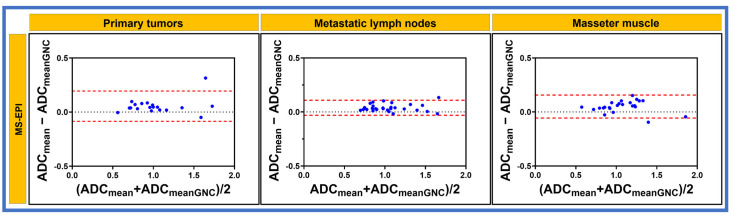
Bland–Altman plot exhibiting agreement between the measurements of mean apparent diffusion coefficient (ADC × 10^−3^ mm^2^/s) values obtained with and without gradient nonlinearity correction (GNC) from multi-shot echo planar imaging for primary tumors, metastatic lymph nodes, and masseter muscle. The difference between the two measurements’ ADC values with and without GNC are on the y-axis, and the mean ADC values are on the x-axis. The dashed lines (red) are the 95% limits of agreement.

**Table 1:** Patient characteristics.

Characteristics	n (%)
Age	
Median(range) 60 (39–87 years)	
Sex	
Male	56 (93.3%)
Female	4 (6.7%)
Clinical stage	
I	5 (8.3%)
II	19 (31.7%)
III	16 (26.7%)
IV	20 (33.3%)
Primary tumor location	
Oropharynx	58 (96.6%)
Larynx	2 (3.4%)

**Table 2:** ADC values (mean ± SD) from PVP Phantom with SS-EPI and MS-EPI.

Method	PVP [%]	Isocenter		|ΔADC| × 10^−3^ mm^2^/s ^1^	|rΔADC| (%) ^2^	Off-Center (12 cm)		|ΔADC| × 10^−3^ mm^2^/s ^1^	|rΔADC| (%) ^2^
		ADC × 10^−3^ (mm^2^/s) with GNC	ADC × 10^−3^ (mm^2^/s) Without GNC			ADC × 10^−3^ (mm^2^/s) with GNC	ADC × 10^−3^ (mm^2^/s) Without GNC		
SS-EPI	40	0.595 ± 0.024	0.598 ± 0.024	0.003 ± 0.001	0.5 ± 0.1	0.622 ± 0.070	0.505 ± 0.060	0.117 ± 0.010	18.8 ± 14.3
	20	1.132 ± 0.055	1.142 ± 0.056	0.010 ± 0.001	0.9 ± 1.8	1.193 ± 0.105	0.992 ± 0.088	0.201 ± 0.017	16.8 ± 16.2
MS-EPI	40	0.625 ± 0.062	0.628 ± 0.062	0.003 ± 0.001	0.5 ± 0.1	0.616 ± 0.042	0.546 ± 0.040	0.070 ± 0.002	11.4 ± 4.8
	20	1.196 ± 0.013	1.199 ± 0.014	0.003 ± 0.001	0.3 ± 7.7	1.227 ± 0.038	0.986 ± 0.040	0.241 ± 0.002	19.6 ± 5.3

^1^ |ΔADC| = |(ADC − ADC_GNC_)|; ^2^ |rΔADC| (%) = |(ADC − ADC_GNC_)/ADC_GNC_| × 100.

**Table 3:** ADC values (mean ± SD, skewness, and kurtosis) were obtained from single-shot (SS) echo planar imaging (EPI) and multi-shot (MS)-EPI data acquisition methods from HNC patients.

Method		Primary Tumor		Metastatic Lymph Nodes		Masseter Muscle	
SS-EPI	Number of patients (n)	38		55		44	
		with GNC	Without GNC	with GNC	Without GNC	with GNC	Without GNC
	(Mean ± SD) × 10^−3^ (mm^2^/s)	0.71 ± 0.14	0.75 ± 0.16 ***	0.84 ± 0.35	0.87 ± 0.37 ***	1.06 ± 0.36	1.15 ± 0.41 ***
	Skewness	0.15 ± 0.44	0.17 ± 0.45 *	0.42 ± 0.74	0.45 ± 0.74 *	−0.27 ± 0.78	−0.24 ± 0.81
	Kurtosis	3.44 ± 1.04	3.48 ± 1.02 *	4.45 ± 1.39	4.47 ± 1.45	4.12 ± 1.50	4.10 ± 1.50
MS-EPI	Number of patients (n)	19		28		24	
	(Mean ± SD) × 10^−3^ (mm^2^/s)	1.00 ± 0.32	1.06 ± 0.34 **	1.04 ± 0.27	1.08 ± 0.28 ***	1.04 ± 0.27	1.09 ± 0.27 ***
	Skewness	0.59 ± 0.55	0.61 ± 0.62	0.35 ± 0.71	0.40 ± 0.66 *	−0.13 ± 0.56	−0.08± 0.56 *
	Kurtosis	3.94 ± 1.70	3.83 ± 1.76	3.88 ± 1.03	3.83 ± 0.90	3.61 ± 1.64	3.72 ± 1.58

Note: Wilcoxon matched-pairs signed-rank test: significantly different (*** *p* < 0.0001, ** *p* < 0.001, and * *p* < 0.050).

**Table 4:** Mean ADC values’ bias and bias 95% confidence interval limit for SS-EPI and MS-EPI.

Method	Region	Bias (Mean ± SD) × 10^−3^ (mm^2^/s)	Bias (95% CI)
SS-EPI	Primary tumors	0.034 ± 0.070	[0.17, −0.10]
	Metastatic lymph nodes	0.032 ± 0.062	[0.15, −0.09]
	Masseter muscle	0.058 ± 0.036	[0.13, −0.01]
MS-EPI	Primary tumors	0.054 ± 0.071	[0.19, −0.09]
	Metastatic lymph nodes	0.039 ± 0.035	[0.11, −0.03]
	Masseter muscle	0.050 ± 0.054	[0.16, −0.06]

## Data Availability

The data presented in this study will be provided upon reasonable request.

## References

[1] Chawla S., Kim S., Wang S., Poptani H. (2009). Diffusion-weighted imaging in head and neck cancers. Future Oncol..

[2] Vandecaveye V., De Keyzer F., Vander Poorten V., Dirix P., Verbeken E., Nuyts S., Hermans R. (2009). Head and neck squamous cell carcinoma: Value of diffusion-weighted MR imaging for nodal staging. Radiology.

[3] Holzapfel K., Duetsch S., Fauser C., Eiber M., Rummeny E.J., Gaa J. (2009). Value of diffusion-weighted MR imaging in the differentiation between benign and malignant cervical lymph nodes. Eur. J. Radiol..

[4] Thoeny H.C. (2011). Diffusion-weighted MRI in head and neck radiology: Applications in oncology. Cancer Imaging.

[5] Dai Y., King A. (2018). State of the art MRI in head and neck cancer. Clin. Radiol..

[6] Le Bihan D. (1990). IVIM method measures diffusion and perfusion. Diagn Imaging.

[7] Padhani A.R., Liu G., Koh D.M., Chenevert T.L., Thoeny H.C., Takahara T., Dzik-Jurasz A., Ross B.D., Van Cauteren M., Collins D. (2009). Diffusion-weighted magnetic resonance imaging as a cancer biomarker: Consensus and recommendations. Neoplasia.

[8] Kato H., Kanematsu M., Tanaka O., Mizuta K., Aoki M., Shibata T., Yamashita T., Hirose Y., Hoshi H. (2009). Head and neck squamous cell carcinoma: Usefulness of diffusion-weighted MR imaging in the prediction of a neoadjuvant therapeutic effect. Eur. Radiol..

[9] Millen R., De Kort W.W.B., Koomen M., van Son G.J.F., Gobits R., Penning de Vries B., Begthel H., Zandvliet M., Doornaert P., Raaijmakers C.P.J. (2023). Patient-derived head and neck cancer organoids allow treatment stratification and serve as a tool for biomarker validation and identification. Med.

[10] Padhani A.R., Koh D.-M. (2011). Diffusion MR imaging for monitoring of treatment response. Magn. Reson. Imaging Clin..

[11] Padhani A.R., Patel S.M. (2011). Diffusion-Weighted Imaging. Clinical MRI of the Abdomen: Why, How, When.

[12] Meyer H.J., Leifels L., Hamerla G., Höhn A.K., Surov A. (2018). ADC-histogram analysis in head and neck squamous cell carcinoma. Associations with different histopathological features including expression of EGFR, VEGF, HIF-1α, Her 2 and p53. A preliminary study. Magn. Reson. Imaging.

[13] Yuan J., Yeung D.K., Mok G.S., Bhatia K.S., Wang Y.X., Ahuja A.T., King A.D. (2014). Non-Gaussian analysis of diffusion weighted imaging in head and neck at 3T: A pilot study in patients with nasopharyngeal carcinoma. PLoS ONE.

[14] Kim S., Loevner L., Quon H., Sherman E., Weinstein G., Kilger A., Poptani H. (2009). Diffusion-weighted magnetic resonance imaging for predicting and detecting early response to chemoradiation therapy of squamous cell carcinomas of the head and neck. Clin. Cancer Res..

[15] Berrak S., Chawla S., Kim S., Quon H., Sherman E., Loevner L.A., Poptani H. (2011). Diffusion weighted imaging in predicting progression free survival in patients with squamous cell carcinomas of the head and neck treated with induction chemotherapy. Acad. Radiol..

[16] Vandecaveye V., Dirix P., De Keyzer F., Op de Beeck K., Vander Poorten V., Hauben E., Lambrecht M., Nuyts S., Hermans R. (2012). Diffusion-weighted magnetic resonance imaging early after chemoradiotherapy to monitor treatment response in head-and-neck squamous cell carcinoma. Int. J. Radiat. Oncol. Biol. Phys..

[17] Chawla S., Kim S.G., Loevner L.A., Wang S., Mohan S., Lin A., Poptani H. (2020). Prediction of distant metastases in patients with squamous cell carcinoma of head and neck using DWI and DCE-MRI. Head. Neck.

[18] van der Hulst H.J., Vos J.L., Tissier R., Smit L.A., Martens R.M., Beets-Tan R.G.H., van den Brekel M.W.M., Zuur C.L., Castelijns J.A. (2022). Quantitative Diffusion-Weighted Imaging Analyses to Predict Response to Neoadjuvant Immunotherapy in Patients with Locally Advanced Head and Neck Carcinoma. Cancers.

[19] Mohamed A.S.R., Abusaif A., He R., Wahid K.A., Salama V., Youssef S., McDonald B.A., Naser M., Ding Y., Salzillo T.C. (2023). Prospective validation of diffusion-weighted MRI as a biomarker of tumor response and oncologic outcomes in head and neck cancer: Results from an observational biomarker pre-qualification study. Radiother. Oncol..

[20] Paudyal R., Oh J.H., Riaz N., Venigalla P., Li J., Hatzoglou V., Leeman J., Nunez D.A., Lu Y., Deasy J.O. (2017). Intravoxel incoherent motion diffusion-weighted MRI during chemoradiation therapy to characterize and monitor treatment response in human papillomavirus head and neck squamous cell carcinoma. J. Magn. Reason. Imaging.

[21] Doblas S., Almeida G.S., Blé F.X., Garteiser P., Hoff B.A., McIntyre D.J., Wachsmuth L., Chenevert T.L., Faber C., Griffiths J.R. (2015). Apparent diffusion coefficient is highly reproducible on preclinical imaging systems: Evidence from a seven-center multivendor study. J. Magn. Reson. Imaging.

[22] Keenan K.E., Peskin A.P., Wilmes L.J., Aliu S.O., Jones E.F., Li W., Kornak J., Newitt D.C., Hylton N.M. (2016). Variability and bias assessment in breast ADC measurement across multiple systems. J. Magn. Reson. Imaging.

[23] Obuchowski N.A., Bullen J. (2018). Quantitative imaging biomarkers: Effect of sample size and bias on confidence interval coverage. Stat. Methods Med. Res..

[24] Glover G.H., Pelc N.J. (1986). Method for Correcting Image Distortion Due to Gradient Nonuniformity. United. States Patent.

[25] Bammer R., Markl M., Barnett A., Acar B., Alley M., Pelc N., Glover G., Moseley M. (2003). Analysis and generalized correction of the effect of spatial gradient field distortions in diffusion-weighted imaging. Magn. Reson. Med..

[26] Janke A., Zhao H., Cowin G.J., Galloway G.J., Doddrell D.M. (2004). Use of spherical harmonic deconvolution methods to compensate for nonlinear gradient effects on MRI images. Magn. Reson. Med..

[27] Malyarenko D.I., Ross B.D., Chenevert T.L. (2014). Analysis and correction of gradient nonlinearity bias in apparent diffusion coefficient measurements. Magn. Reson. Med..

[28] Tan E.T., Marinelli L., Slavens Z.W., King K.F., Hardy C.J. (2013). Improved correction for gradient nonlinearity effects in diffusion-weighted imaging. J. Magn. Reson. Imaging.

[29] Tao S., Trzasko J.D., Gunter J.L., Weavers P.T., Shu Y., Huston J., Lee S.K., Tan E.T., Bernstein M.A. (2017). Gradient nonlinearity calibration and correction for a compact, asymmetric magnetic resonance imaging gradient system. Phys. Med. Biol..

[30] Mesri H.Y., David S., Viergever M.A., Leemans A. (2020). The adverse effect of gradient nonlinearities on diffusion MRI: From voxels to group studies. NeuroImage.

[31] Pang Y., Malyarenko D.I., Amouzandeh G., Barberi E., Cole M., Vom Endt A., Peeters J., Tan E.T., Chenevert T.L. (2021). Empirical validation of gradient field models for an accurate ADC measured on clinical 3T MR systems in body oncologic applications. Phys. Med..

[32] Malyarenko D.I., Newitt D.C., Amouzandeh G., Wilmes L.J., Tan E.T., Marinelli L., Devaraj A., Peeters J.M., Giri S., Vom Endt A. (2020). Retrospective correction of ADC for gradient nonlinearity errors in multicenter breast DWI trials: ACRIN6698 multiplatform feasibility study. Tomography.

[33] Konar A.S., Fung M., Paudyal R., Oh J.H., Mazaheri Y., Hatzoglou V., Shukla-Dave A. (2020). Diffusion-Weighted Echo Planar Imaging using MUltiplexed Sensitivity Encoding and Reverse Polarity Gradient in Head and Neck Cancer: An Initial Study. Tomography.

[34] Tamada T., Kido A., Ueda Y., Takeuchi M., Kanki A., Neelavalli J., Yamamoto A. (2022). Comparison of single-shot EPI and multi-shot EPI in prostate DWI at 3.0 T. Sci. Rep..

[35] Chen H., Dai K., Zhong S., Zheng J., Zhang X., Yang S., Cao T., Wang C., Karasan E., Frydman L. (2023). High-resolution multi-shot diffusion-weighted MRI combining markerless prospective motion correction and locally low-rank constrained reconstruction. Magn. Reson. Med..

[36] Dong Y., Riedel M., Koolstra K., van Osch M.J.P., Börnert P. (2023). Water/fat separation for self-navigated diffusion-weighted multishot echo-planar imaging. NMR Biomed..

[37] Amouzandeh G., Chenevert T.L., Swanson S.D., Ross B.D., Malyarenko D.I. (2022). Temperature and concentration dependence of water diffusion in polyvinylpyrrolidone solutions. Med. Phys..

[38] LoCastro E., Paudyal R., Konar A.S., LaViolette P.S., Akin O., Hatzoglou V., Goh A.C., Bochner B.H., Rosenberg J., Wong R.J. (2023). A Quantitative Multiparametric MRI Analysis Platform for Estimation of Robust Imaging Biomarkers in Clinical Oncology. Tomography.

[39] Stejskal E.O., Tanner J.E. (1965). Spin diffusion measurements: Spin echoes in the presence of a time-dependent field gradient. J. Chem. Phys..

[40] King A.D., Chow K.K., Yu K.H., Mo F.K., Yeung D.K., Yuan J., Bhatia K.S., Vlantis A.C., Ahuja A.T. (2013). Head and neck squamous cell carcinoma: Diagnostic performance of diffusion-weighted MR imaging for the prediction of treatment response. Radiology.

[41] Surov A., Meyer H.J., Wienke A. (2017). Correlation between apparent diffusion coefficient (ADC) and cellularity is different in several tumors: A meta-analysis. Oncotarget.

[42] Habrich J., Boeke S., Fritz V., Koerner E., Nikolaou K., Schick F., Gani C., Zips D., Thorwarth D. (2024). Reproducibility of diffusion-weighted magnetic resonance imaging in head and neck cancer assessed on a 1.5 T MR-Linac and comparison to parallel measurements on a 3 T diagnostic scanner. Radiother. Oncol..

[43] Lemainque T., Yoneyama M., Morsch C., Iordanishvili E., Barabasch A., Schulze-Hagen M., Peeters J.M., Kuhl C., Zhang S. (2024). Reduction of ADC bias in diffusion MRI with deep learning-based acceleration: A phantom validation study at 3.0 T. Magn. Reson. Imaging.

[44] Widmann G., Henninger B., Kremser C., Jaschke W. (2017). MRI sequences in head & neck radiology–state of the art. RöFo-Fortschritte auf dem Gebiet der Röntgenstrahlen und der Bildgebenden Verfahren.

[45] Liao C., Cao X., Cho J., Zhang Z., Setsompop K., Bilgic B. Highly efficient MRI through multi-shot echo planar imaging. Proceedings of the Wavelets and Sparsity XVIII.

[46] Aggarwal H.K., Mani M.P., Jacob M. Multi-shot sensitivity-encoded diffusion MRI using model-based deep learning (MODL-MUSSELS). Proceedings of the 2019 IEEE 16th International Symposium on Biomedical Imaging (ISBI 2019).

[47] Kim Y.-Y., Kim M.-J., Gho S.-M., Seo N. (2020). Comparison of multiplexed sensitivity encoding and single-shot echo-planar imaging for diffusion-weighted imaging of the liver. Eur. J. Radiol..

[48] Altmann S., Mercado M.A.A., Brockstedt L., Kronfeld A., Clifford B., Feiweier T., Uphaus T., Groppa S., Brockmann M.A., Othman A.E. (2023). Ultrafast brain MRI protocol at 1.5 T using deep learning and multi-shot EPI. Acad. Radiol..

[49] Chen H., Tang R., Song X., Zong R., Liu J., Jin C., Deng K. (2024). Comparison of single shot and multishot diffusion-weighted imaging in 5-T magnetic resonance imaging for brain disease diagnosis. Quant. Imaging Med. Surg..

[50] Chen N.K., Guidon A., Chang H.C., Song A.W. (2013). A robust multi-shot scan strategy for high-resolution diffusion weighted MRI enabled by multiplexed sensitivity-encoding (MUSE). Neuroimage.

[51] Pruessmann K.P., Weiger M., Scheidegger M.B., Boesiger P. (1999). SENSE: Sensitivity encoding for fast MRI. Magn. Reson. Med..

[52] Aliotta E., Paudyal R., Dresner A., Shukla-Dave A., Lee N., Cerviño L., Otazo R., Yu V.Y. (2024). Reduced-distortion diffusion weighted imaging for head and neck radiotherapy. Phys. Imaging Radiat. Oncol..

[53] Skare S., Newbould R.D., Clayton D.B., Albers G.W., Nagle S., Bammer R. (2007). Clinical multishot DW-EPI through parallel imaging with considerations of susceptibility, motion, and noise. Magn. Reson. Med..

[54] Soher B.J., Dale B.M., Merkle E.M. (2007). A Review of MR Physics: 3T versus 1.5T. Magn. Reson. Imaging Clin. N. Am..

[55] Srinivasan A., Dvorak R., Perni K., Rohrer S., Mukherji S. (2008). Differentiation of benign and malignant pathology in the head and neck using 3T apparent diffusion coefficient values: Early experience. Am. J. Neuroradiol..

[56] Lavdas I., Miquel M.E., McRobbie D.W., Aboagye E.O. (2014). Comparison between diffusion-weighted MRI (DW-MRI) at 1.5 and 3 tesla: A phantom study. J. Magn. Reson. Imaging.

